# Modelling Impact of Site and Terrain Morphological Characteristics on Biomass of Tree Species in Putorana Region

**DOI:** 10.3390/plants10122722

**Published:** 2021-12-10

**Authors:** Ján Merganič, Viliam Pichler, Erika Gömöryová, Peter Fleischer, Marián Homolák, Katarína Merganičová

**Affiliations:** 1 Faculty of Forestry, Technical University in Zvolen, T. G. Masaryka, 96001 Zvolen, Slovakia; pichler@tuzvo.sk (V.P.); gomoryova@tuzvo.sk (E.G.); p.fleischersr@gmail.com (P.F.); marian.homolak@tuzvo.sk (M.H.); 2 Faculty of Forestry and Wood Sciences, Czech University of Life Sciences Prague, Kamýcká 129, 6-Suchdol, 16500 Praha, Czech Republic; merganicova@fld.czu.cz or; 3 Department of Biodiversity of Ecosystems and Landscape, Institute of Landscape Ecology, Slovak Academy of Sciences, Akademická 2, 94901 Nitra, Slovakia

**Keywords:** boreal forests, height-diameter models, crown radius-height models, deadwood, subarctic zone, Siberia

## Abstract

(1) Background: Boreal forests influence global carbon balance and fulfil multiple ecosystem services. Their vegetation growth and biomass are significantly affected by environmental conditions. In the present study we focused on one of the least accessible and least studied parts of the boreal region situated in the western part of Putorana plateau, Central Siberia (Lama and Keta lakes, Krasnoyarsk region), northern Russia. (2) Methods: We derived local height-diameter and crown radius-height models for six tree species. We used univariate correlation and multiple regression analyses to examine the relationships between tree biomass and environmental conditions. (3) Results: Total tree biomass stock (aboveground tree biomass + aboveground and buried deadwood) varied between 6.47 t/ha and 149 t/ha, while total deadwood biomass fluctuated from 0.06 to 21.45 t/ha. At Lama, biomass production decreased with elevation. At Keta, the relationship of biomass to elevation followed a U shape. Stand biomass changed with micro-terrain morphology and soil nutrient content, while the patterns were location-specific. (4) Conclusions: The majority of the derived models were significant and explained most of the variability in the relationships between tree diameter or crown radius and tree height. Micro-site environmental conditions had a substantial effect on tree biomass in the studied locations.

## 1. Introduction

Boreal forests, also called taiga, are the Earth’s northernmost forests that spread across northern Europe, Russia, Alaska, and Canada. They provide a valuable habitat for wildlife and multiple ecosystem services, including carbon storage and clean water [1]. However, climate change has recently been causing rapid changes in these high-latitude ecosystems [2]. This will significantly influence the global carbon balance, although it is still not clear if boreal forests will be a net carbon sink or source in the future due to the global warming [3]. Changes in environmental conditions will significantly affect vegetation biomass [4,5], which is an important characteristic of forest ecosystems [6] from the point of carbon sequestration, and hence of global carbon budget.

Approximately 27% of global aboveground biomass and 50% of organic soil carbon is stored in boreal forests [7]. The forests there sequester approximately one fifth of the total C sink generated by the world’s forests [8]. Although they represent very precious ecosystems, many of their parts have not been scientifically studied due to their inaccessibility. Thus, understanding of the processes and relationships between vegetation and environment in such hardly accessible ecosystems is needed. Such knowledge may help to reduce the impact of global warming due to the greenhouse effect [9].

In spite of fast development of remote sensing methods for the estimation of biomass over large and inaccessible areas [10], field measurements remain an important source of information. Tree diameter and height are two basic characteristics that describe trees and forest stands and are used to estimate tree volume [11]. Accurate measurements of diameter and height are required to derive growth and yield models used in forest management planning [12]. Since tree heights are usually more difficult and/or more demanding to measure in the field, height-diameter models are frequently derived from a subset of trees at plots to estimate heights of the trees, for which only diameter was acquired. Moreover, height-diameter functions help the evaluation of site productivity in uneven-aged and mixed-species stands [11,13,14,15]. Previous studies showed that height-diameter relationships depend on environmental conditions [12]. Regional differences in height-diameter relationships for different parts of the world were found by [11,16,17]. These studies show that region-specific models are of a grea*t* value for correct estimation of stand volume and biomass.

Above-ground biomass (AGB) is the most visible carbon pool of vegetation, which has received most of scientific attention. Its changes indicate shifts due to growth, disturbances, and changes in environmental conditions that affect carbon sequestration [18]. Direct measurements of AGB can be performed only with destructive harvesting. This approach is expensive and time-consuming, which keeps the number of measured trees low [19]. The information gathered from the sampled trees represents a basic dataset to derive size-mass allometric relationships based on more easily measured parameters [3,19]. Although generalised biomass models are available to estimate forest biomass stock [20], IPCC recommends applying locally fitted models to minimise the estimation bias [21]. Tree diameter at breast height (DBH) is the most commonly used predictor of biomass, because of its explanatory power for the estimation of total, subtotal, or component biomass [6,22,23,24]. However, DBH is usually used if it exceeds certain thresholds, usually 7 cm, but smaller thresholds of 3 to 5 cm have also been applied [25,26]. In unmanaged boreal forests, a substantial part of the total aboveground carbon is stored in thin and small trees and shrubs [2]. The best predictors of aboveground biomass of such short-stature shrubs and trees have been the stem base diameter [27,28,29,30,31], tree height [32,33,34,35], crown width, or crown area [34,36]. Models obtain either a single independent variable, or multiple variables [37,38].

The present study focuses on the northern part of Russia, one of the least accessible and least studied regions of the country. To the best of our knowledge, this study is the first one focusing on the area around Keta lake and its watershed. In the area around the Lama lake, only a few studies [39,40,41] dealing with the upward shift of the upper timber line have been conducted. The forests there are mixed, composed of broadleaved and coniferous species including *Larix spp.*, which dominates many forests of the global circumpolar boreal region and is the most wide-spread genus in Russia. In addition to larch, *Picea obovata* L. is another productive species in Putorana forests [42]. The main objective of this article is to analyse the impact of environmental conditions on the accumulated biomass of tree species in the western part of Putorana Plateau in the area around the Lama and Keta lakes (Krasnoyarsk region, Russia). Partial objectives were to: (1) develop local height-diameter models and (2) crown radius height models for six tree species along elevational gradients; (3) and to derive the above-ground biomass from dendrometric measurements using regional allometric equations by [38,43,44].

## 2. Results

The stand density of the examined forest stands changed with location and elevation. At Lama location, the stand density of living trees was the highest (9460 pcs/ha) in the highest elevational zone (419 m a.s.l.) similarly as in the work of [41]. The density at the lowest elevational zone (129 m a.s.l.) was by 1.37 times lower. However, the lowest stand density was observed in the middle elevational zone (249 m a.s.l.). The least dense stands in the middle elevational zone were also recorded at the Keta location. However, the maximum stand density at Keta was observed in the lowest zone (104 m a.s.l.), where the canopies of the main tree layer were opened, and the understorey was dense.

### 2.1. Height-Diameter Models

**Figure 1:**
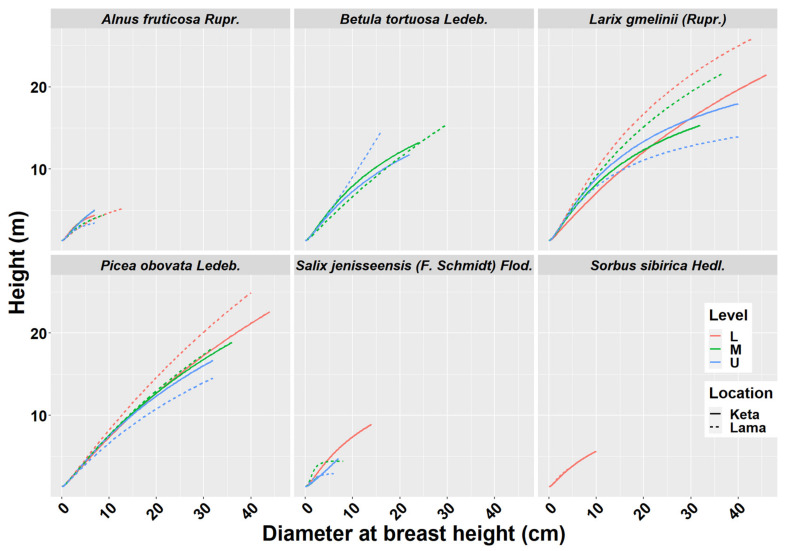
Height-diameter models of six tree species at two locations of Keta and Lama. Statistical parameters of models are presented in Table 1. Level—L—lower elevational zone, M—middle elevational zone, U—upper elevational zone.

We derived height-diameter models separately for six tree species, two locations (Keta and Lama) and three elevational zones separately. In total we derived 29 models, because not all species occurred at all elevational zones. As can be seen in Figure 1, the position and the shape of the height-diameter models is influenced by the location and the factors aggregated in the elevational gradient. The models derived for the Lama location follow the logical pattern along the elevational gradient, i.e., tree dimensions were largest in the lowest zone and decreased with the increasing elevation. This pattern is visible mainly for *Larix gmelinii* (Rupr.), *Picea obovata* Ledeb., *Salix jenisseensis* (F. Schmidt) Flod., and partly also for *Alnus fruticosa* Rupr. The height-diameter curves of *Betula tortuosa* Ledeb. revealed that higher trees occurred in the upper elevational zones. In the case of *Alnus fruticosa* Rupr. we did not find any difference between the lower and middle zones. At Keta, differences between elevational zones were smaller in comparison with the Lama location. The pattern for *Larix gmelinii* (Rupr.) indicates that other factors are more influential than the elevational gradient. The majority of the derived models were significant and explained most of the variability in the relationship between diameter and height (Table 1). The lowest and the highest correlations were found for *Salix jenisseensis* (F. Schmidt) Flod. (R^2^ between 0.14 and 1). Models for other species explained approximately 90% of variability, as their R^2^ fluctuated from 0.88 to 0.99 depending on the species, location, and the elevational zone (Table 1). Statistical comparison of models between elevational zones revealed that models at Keta are more similar than those at Lama (Figure A2). Significantly different height-diameter models between locations were found for all tree species except for *Salix*, while the inter-location differences were most pronounced for *Alnus* (Figure A2).

**Figure A1:**
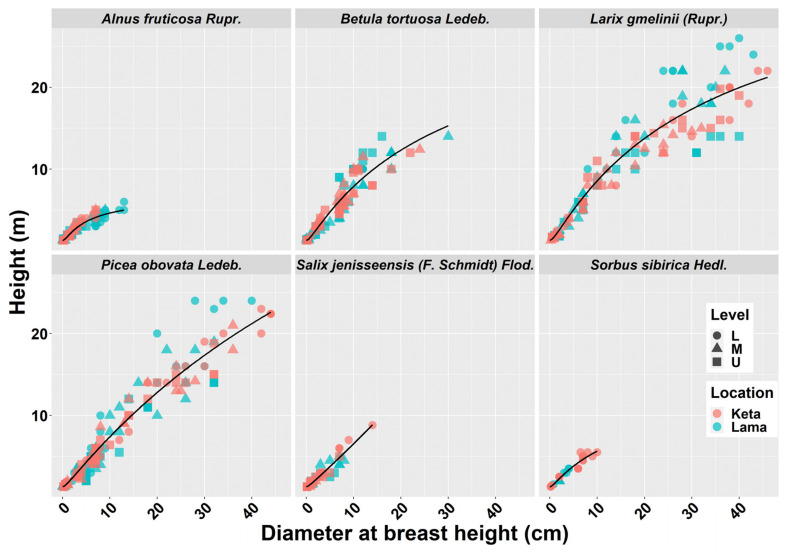
General height-diameter models for six tree species at two locations of Keta and Lama. Statistical parameters of models are presented in Table 1. Level: L—lower elevational zone, M—middle elevational zone, U—upper elevational zone.

We also derived general species-specific models for both locations together regardless of elevation (Table 1, Figure A1). These models were highly significant and explained 88% to 93% of variability in height-diameter relationships of individual species.

### 2.2. Crown Radius—Height Models

**Figure 2:**
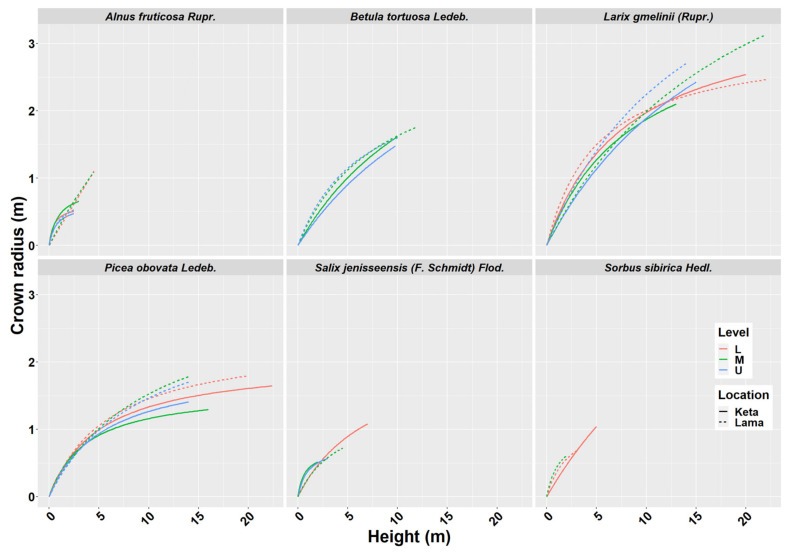
Crown radius—height models of six tree species at two locations of Keta and Lama. Statistical parameters of models are presented in Table 2. Level: L—lower elevational zone, M—middle elevational zone, U—upper elevational zone.

**Figure A2:**
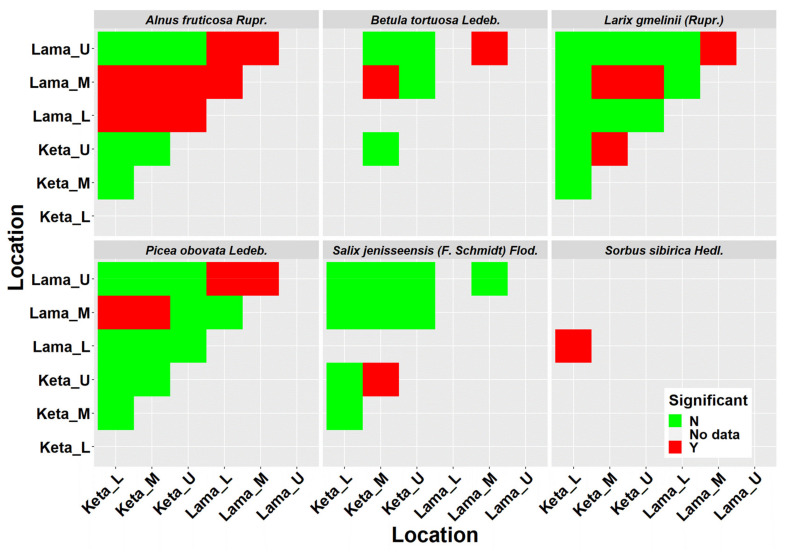
ANOVA-based statistical comparison of derived height-diameter models (Table 1). N means not significant, Y means significant at 95% significance level. L, M, U represent lower, middle, and upper elevational zones, respectively.

Crown width has an important role in many biomass models. From the obtained data we derived 29 models describing the relationship of crown radius to tree height at the individual location and elevational category (Figure 2). The results revealed a lower impact of the elevational gradient than the one found for height-diameter models. In the case of *Alnus fruticosa* Rupr., the statistical test did not confirm significant differences between the elevational zones at the Keta location (Figure A2). At the Lama location, we found a significant difference between the upper and the middle zones, as well as between the upper and the lower zones. The relationship between the crown radius and tree height in the lower and middle zones of Lama has a linear pattern. The relationships in the upper zone are non-linear and similar at both locations. The models of individual locations significantly differed from each other (Figure A2).

The models derived for *Betula tortuosa* Ledeb. at Keta significantly differed between the elevational zones, while at Lama we did not reveal such an influence of elevation. Moreover, the models derived for locations were significantly different. In the case of *Larix gmelinii* (Rupr.), we did not reveal any significant differences between the models of individual elevational zones, but we confirmed significant differences between the locations. The models for *Picea obovata* Ledeb. significantly differed between the elevational zones at Keta, while the lowest crown radii were found in the middle elevational zone, and the largest ones in the lower zone. At Lama, the significant difference was found between the models for the lower and the middle zones, which were characterised by the lowest and the largest crown radii, respectively. The models significantly differed between locations. For *Salix jenisseensis* (F. Schmidt) Flod. we did not find significant differences between elevational zones at Keta. At Lama we could derive only one model for the middle elevational zone. The models for individual locations were not significantly different. For *Sorbus sibirica* Hedl. we created only three models in total, one for the lower elevational zone at Keta, and the other two for two elevational zones at Lama, which did not differ from each other significantly. However, the models for Keta and Lama were significantly different. Parameters of crown radius-height models for individual tree species, elevational zones, and locations are presented in Table 2. In general, the models explained from 58% to 99% of crown radius variability depending on the tree species, location, and elevational zone (Table 2).

**Figure A3:**
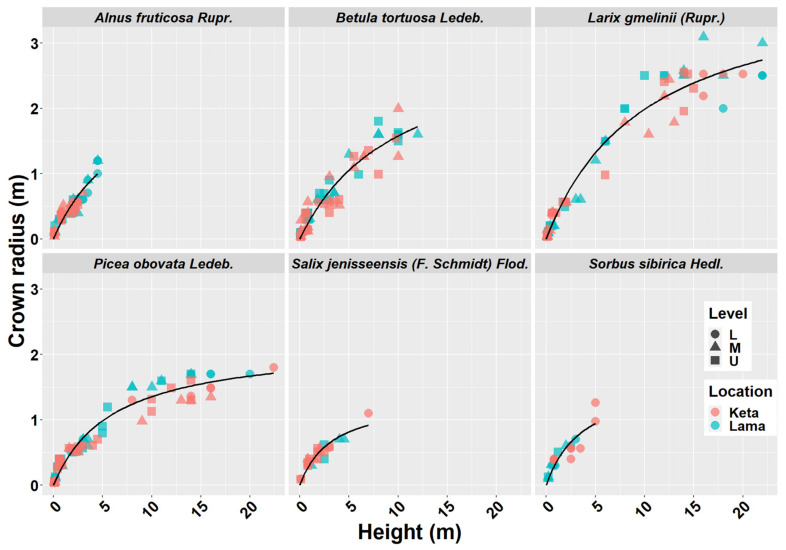
General crown radius, height models for six tree species at two locations of Keta and Lama. Statistical parameters of models are presented in Table 2. Level: L—lower elevational zone, M—middle elevational zone, U—upper elevational zone.

General species-specific models explained between 79% and 96% of variability in examined relationships (Table 2, Figure A3).

### 2.3. Above-Ground Biomass (AGB)

**Figure 3:**
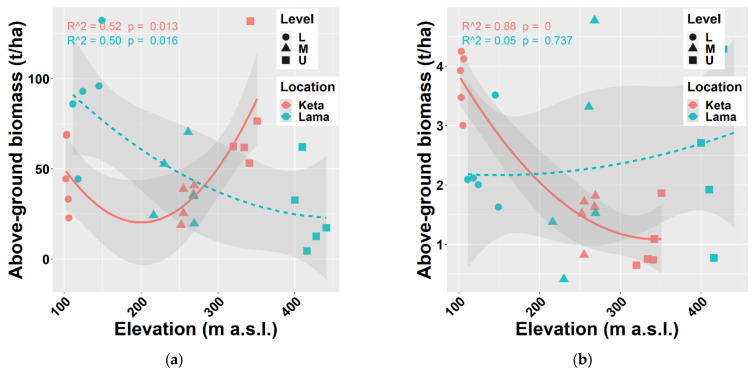
Relationship between elevation and aboveground living biomass (**a**) of all trees together, (**b**) of thin trees (with a height of at least 0.1 m up to DBH ≤ 7 cm) at two locations. Points represent biomass values at individual sample plots. Lines represent fitted non-linear models, and grey areas show 95% confidence intervals around the fitted models. Level: L—lower elevational zone, M—middle elevational zone, U—upper elevational zone.

The biomass of tree species was calculated using regional allometric equations derived by [38,43,44] for thick and thin trees separately. In general, biomass production decreases with elevation. We found this pattern at Lama, where the aboveground biomass of all tree species decreased from 90 t/ha to 26 t/ha, i.e., by 3.5 times along the elevational gradient covering 300 m. At Keta, the decreasing trend was recorded only up to middle elevations (206 m a.s.l.). At higher elevations, biomass stock increased and reached the value of about 77 t/ha at the highest elevation. The aboveground biomass of thin trees (DBH ≤ 7 cm) was slightly increasing with the elevation at Lama, while at Keta we found a substantial decreasing trend (Figure 3).

**Figure 4:**
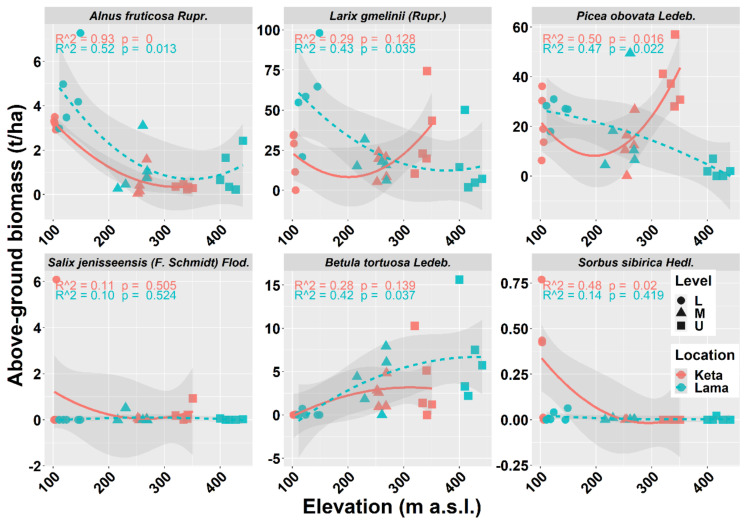
Relationship between elevation and aboveground living tree biomass of individual tree species at two locations. Points represent biomass values at individual sample plots. Lines represent fitted non-linear models, and grey areas show 95% confidence intervals around the fitted models. Level: L—lower elevational zone, M—middle elevational zone, U—upper elevational zone.

The analysis of individual tree species revealed that the aboveground biomass of *Alnus fruticosa* Rupr. decreased with elevation at both locations. In contrast, the aboveground tree biomass of *Betula tortuosa* Ledeb. increased with elevation at either of the studied locations. In the case of *Salix jenisseensis* (F. Schmidt) Flod. and *Sorbus sibirica* Hedl. we observed a decrease in aboveground biomass with elevation at Keta, while at Lama we did not find any strong trend with elevation, and the aboveground biomass remained unchanged. The relationship between the elevation and the biomass of the main tree species *Larix gmelinii* (Rupr.) and *Picea obovata* Ledeb. resembles the general pattern of all tree species together (compare Figure 3 and Figure 4).

**Figure 5:**
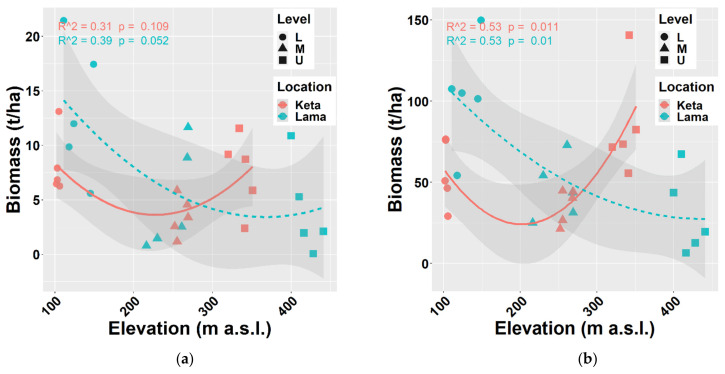
Relationships between elevation and biomass of deadwood (**a**) and total biomass (living and deadwood together) (**b**) at two locations. Points represent biomass values at individual sample plots. Lines represent fitted non-linear models, and grey areas show 95% confidence intervals around the fitted models. Level: L—lower elevational zone, M—middle elevational zone, U—upper elevational zone.

Next, we looked at the relationship between elevation and deadwood biomass that included both fine and coarse woody debris (stumps, standing dead trees, snags, logs, and buried deadwood) (Figure 5a) and the total biomass, i.e., living and dead together (Figure 5b). The revealed trends are similar to the one of the aboveground living biomass of all tree species or main tree species presented above. Total deadwood biomass at individual sample plots varied between 0.06 t/ha and 21.45 t/ha, while the recorded total biomass stock was from 6.47 t/ha to 149 t/ha.

### 2.4. Relationships of Selected Site Characteristics to Biomass of Tree Species

We performed a univariate correlation and regression analysis to examine the relationship of 99 environmental variables (see Figure A5, and Materials and Methods for more details) to the total biomass of tree species (living and dead together) in investigated locations. In total, we found 10 significant relationships at 95% significance level (Figure A5). Only one of them (biomass versus amount of K in mg/kg in the upper soil layer up to 10 cm depth) was significant at 99% significance level (R^2^ = 0.44). Out of 10 significant correlations, six were negative and four positive ones. The total biomass was significantly positively correlated with terrain types (represented in models as dummy variables), where water inflow exceeds water outflow from a site, such as a slope base, a bottom of the slope, a terrace gutter, and a slope basin. Positive relationships were also revealed between biomass and K and Mn content in the upper soil layer up to 10 cm, and the C:N ratio in the soil layer in the depth of 20–30 cm. Negative relationships were revealed between the biomass and the terrain type, where water outflow prevails, e.g., a summit, or an upper part of a slope. The decrease in biomass was also found with the increase in d15N, Al-d, Al-o in the upper soil layer (0–10 cm), and the proportion of hygroscopic water in the second and third soil layers (10–20 or 20–30 cm).

**Figure 6:**
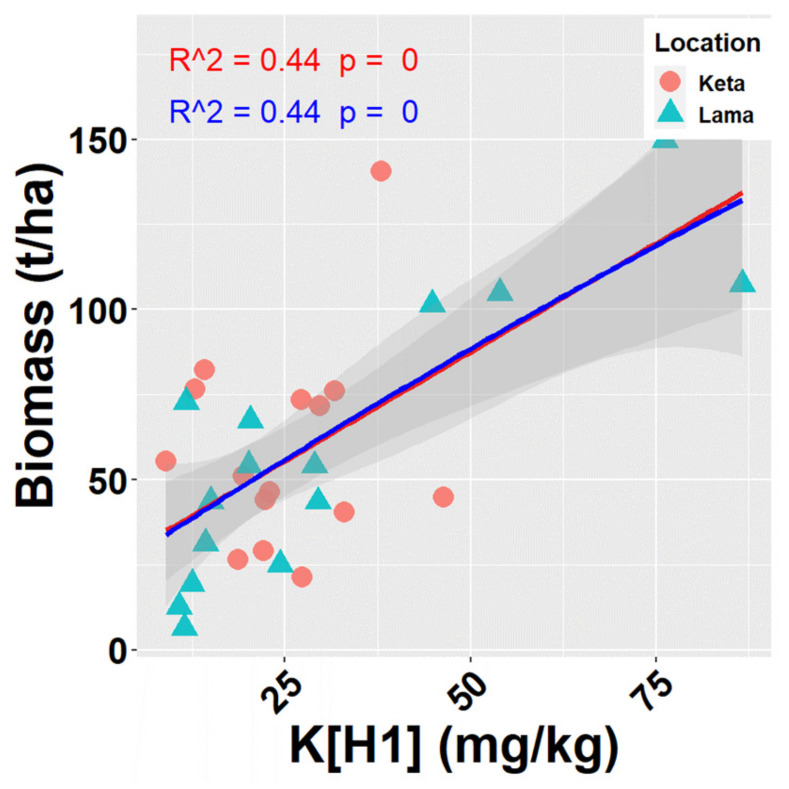
Relationships of K content in soil in depth 0–10 cm (K[H1] (mg/kg)) to total biomass of tree species (aboveground living + deadwood). Points represen*t* values at individual sample plots. Lines represent fitted models (red—linear, blue—quadratic), and grey areas show 95% confidence intervals around the fitted models.

When analysing the relationships of site characteristics to total biomass (living and dead together) for individual locations separately, no significant correlation was revealed at Keta. On the contrary, 28 significant relationships (Figure A5) were found at Lama, where the elevational gradient was more pronounced in previous analyses. Seven of them were positive and the remaining 21 were negative. R^2^ of significant relationships fluctuated between 0.27 and 0.72. The highest correlation was found between total biomass and K content in the first soil layer (0–10 cm) (Figure 6).

The analysis of the impact of site characteristics on the aboveground biomass of living trees revealed similar results for the total biomass. The analysis regardless of location found 6 characteristics significantly affecting aboveground living biomass, while for Keta no significant correlation was revealed, and for Lama 27 significant relationships were detected.

**Figure 7:**
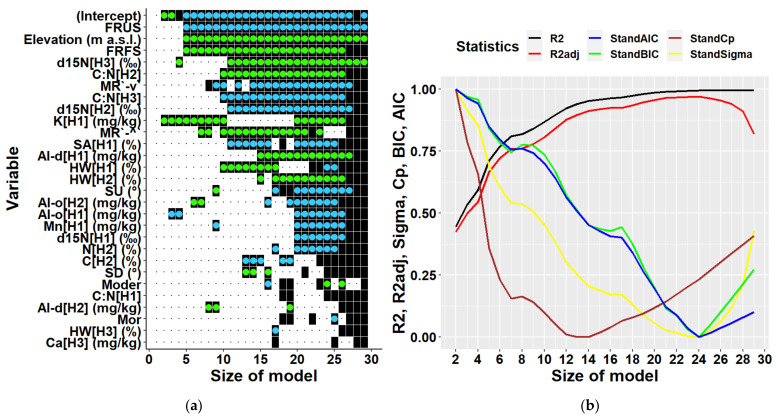
Predictors (**a**) and statistical characteristics (**b**) of multiple regression models of tree biomass derived for both locations together. Size of model represents the number of predictors included in the multiple regression model. Participation of individual variables in multiple regression models of biomass is indicated in the left figure by black squares, while black dots indicate their absence. Green dots inside black squares indicate significant positive impacts of respective variables, while blue dots represent their significant negative impacts. Predictors are identified by a combination of variable abbreviations, soil layers, and units if applicable as follows: Variables: Al-d—content of dithionite-extractable aluminium in (mg/kg), Al-o—content of oxalate-extractable aluminium in (mg/kg), C—carbon content in (%), C:N—C:N ratio, Ca—calcium content in (mg/kg), d15N—ratio of stable nitrogen isotopes 15N:14N in (‰), Elevation—elevation in (m a.s.l.), FRFS—form of relief—foot of slope, FRUS—form of relief—peak, upper slope, HW—hygroscopic water in (%), K—potassium content in (mg/kg), Mn—manganese content in (mg/kg), Moder—humus form, Mor—humus form, MR`-^`—micro relief (flat terrain along the contour line and concave terrain along the slope), MR`-v`—micro relief (flat terrain along the contour line and convex terrain along the slope), N—nitrogen content in (%), SA—sand fraction in (%), SD—slope downward in (°), SU—slope upward (°); soil layers presented in square brackets: H1—layer at 0–10 cm soil depth, H2—10–20 cm depth, H3—20–30 cm depth; statistical characteristics of derived models presented in figure (**b**) are as follows: R^2^—R-squared, R^2^adj—adjusted R-squared, AIC—AKAIKE information criterion, BIC—BAYESIAN information criterion, Cp—Mallows’ statistic, Sigma—residual standard deviation.

Multiple regression analyses of all data together regardless of location partially confirmed results of the univariate correlation analysis (Figure 7). Considering the existing data structure, a multiple regression model can include 28 variables at maximum. Most frequently included variables were elevation and the two terrain types mentioned above. These were followed by soil characteristics, namely d15N in the third soil layer, and C:N ratios in the third and the second soil layers, i.e., in depths of 20–30, and 10–20 cm, respectively. The next commonly included variable was K content in the first soil layer, which was found to be the most highly correlated variable to biomass in univariate analyses. Further variables represented site characteristics (forms of micro-relief and humus, and slope), and other soil characteristics (content of sand, hygroscopic water, content of chemical elements Mn, Ca, and of oxalate- and dithionite-extractable Al). Statistical parameters of multiple models (Figure 7) indicated that the optimal multivariate model should include 12 to 14 predictors (Cp). Models with this number of independent variables can explain 87 to 91% of biomass variability. The model with 13 predictors including an intercept is presented in Table 3. The results show that all included variables were significant, and their multicollinearity did not exceed recommended thresholds, i.e., their values of the variance inflation factor was below 10 [45,46,47]. The types of terrain were the most influential variables in the model (Table 3).

**Figure 8:**
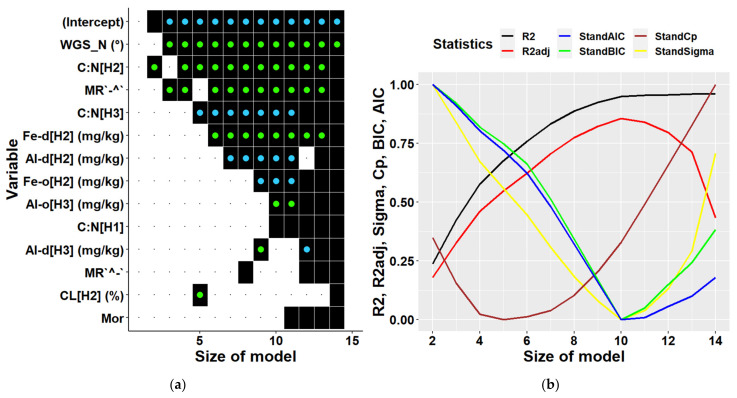
Predictors (**a**) and statistical characteristics (**b**) of multiple regression models of tree biomass derived for Keta location. Size of model represents the number of predictors included in the multiple regression model. Participation of individual variables in multiple regression models of biomass is indicated in the left figure by black squares, while black dots indicate their absence. Green dots inside black squares indicate significant positive impacts of respective variables, while blue dots represent their significant negative impacts. Predictors are identified by a combination of variable abbreviations, soil layers, and units if applicable as follows: Variables: Al-d—content of dithionite-extractable aluminium in (mg/kg), Al-o—content of oxalate-extractable aluminium in (mg/kg), C:N—C:N ratio, CL—clay fraction in (%), Fe-d—content of dithionite-extractable iron in (mg/kg), Fe-o—content of oxalate-extractable iron in (mg/kg), MR`^-`—micro relief (concave terrain along the contour line and flat terrain along the slope), MR`-^`—Micro relief (flat terrain along the contour line and concave terrain along the slope), WGS_N—latitude in (°); soil layers presented in square brackets: H1—layer at 0–10 cm soil depth, H2—10–20 cm depth, H3—20–30 cm depth; statistical characteristics of derived models presented in figure (**b**) are as follows: R^2^—R-squared, R^2^adj—adjusted R-squared, AIC—Akaike information criterion, BIC—Bayesian information criterion, Cp—Mallows’ statistic, Sigma—residual standard deviation.

When deriving multiple regressions for individual locations we could include only 14 predictors at maximum due to a smaller amount of available data. Most of the variables selected as predictors were soil characteristics. The optimal model for Keta (Figure 8) contained 4 to 6 independent variables (including an intercept). The models of these dimensions explained 46–62% of biomass variability. The most significant variables were latitude, C:N ratio in different soil layers, forms of micro-relief (plain along the contour line, concave along the slope). The 5-dimensional model also included the fraction of clay in 10–20 cm soil depth (Table 4).

**Figure 9:**
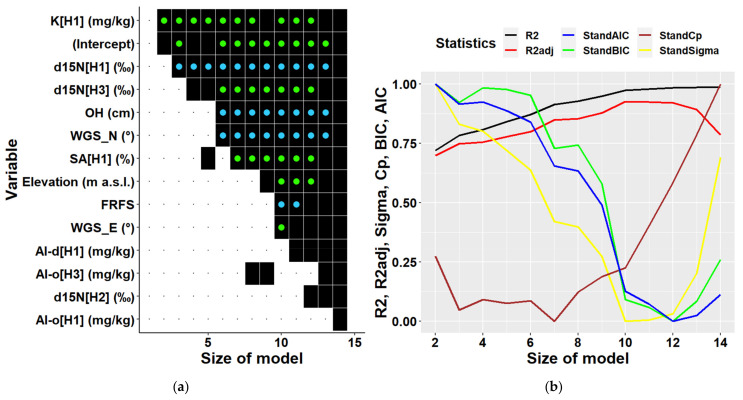
Predictors (**a**) and statistical characteristics (**b**) of multiple regression models of tree biomass derived for Lama location. Size of model represents the number of predictors included in the multiple regression model. Participation of individual variables in multiple regression models of biomass is indicated by black squares, while black dots indicate their absence. Green dots inside black squares indicate significant positive impacts of respective variables, while blue dots represent their significant negative impacts. Predictors are identified by a combination of variable abbreviations, soil layers, and units if applicable as follows: Variables: Al-d—content of dithionite-extractable aluminium in (mg/kg), Al-o—content of oxalate-extractable aluminium in (mg/kg), d15N—ratio of stable nitrogen isotopes 15N:14N in ‰, Elevation—elevation in (m a.s.l.), FRFS—form of relief (foot of slope), K—potassium content in (mg/kg), OH—thickness of O horizon—surface organic layer in (cm), SA—sand fraction in (%), WGS_E—longitude in (°), WGS_N—latitude in (°). Soil layers presented in square brackets: H1—layer at 0–10 cm soil depth, H2—10–20 cm depth, H3—20–30 cm depth. Statistical characteristics of derived models presented in figure (**b**) are as follows: R^2^—R-squared, R^2^adj—adjusted R-squared, AIC—Akaike information criterion, BIC—Bayesian information criterion, Cp—Mallows’ statistic, Sigma—residual standard deviation.

The optimal multiple model for Lama contained 7 variables (Figure 9) and explained 85% of biomass variability (Table 5). The most frequently included variable was K content in soil depth of 0–10 cm. The content of nitrogen isotope d15N in the top (0–10 cm) and bottom (20–30 cm) soil layers were other frequent significant characteristic in multiple models. The other variables were latitude, thickness of O horizon, and the fraction of sand in 0–10 cm soil depth.

## 3. Discussion

Height-diameter models are useful tools for the estimation of tree heights from their diameters as well as for the evaluation of site productivity [11,13,14,15]. A number of different equations have been used to describe the relationship between diameter and height. We used a model by [48,49], because this function was able to describe the relationship between the tree diameter and height of thin trees. The suitability of the specific equation depends on environmental conditions [12]. For example, [17] found that the Chapman–Richards function was best able to describe their empirical data for Dahurian larch, while [16] revealed that the most suitable function changed between the ecoregions of the Daxing’an Mountains Region. Our results also showed differences between locations and elevational zones, although not all of them were significant (Figure A2). However, for each tree species we found some significant differences either between locations or elevational zones or both.

**Figure A4:**
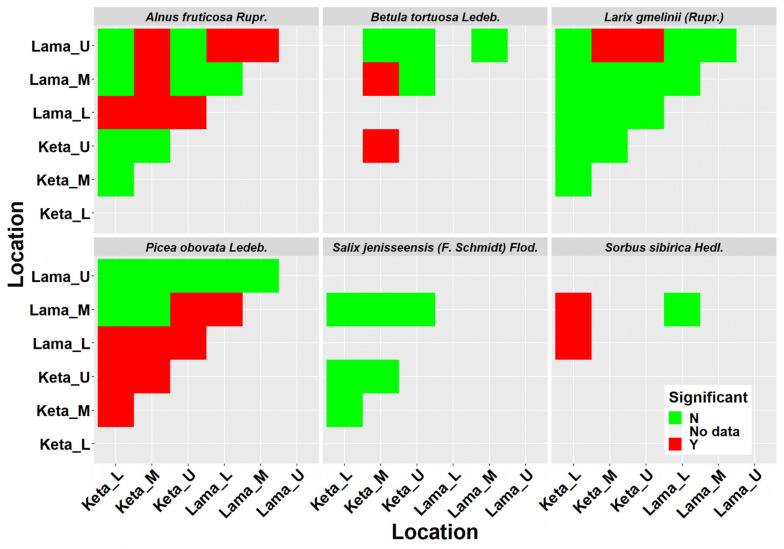
ANOVA-based statistical comparison of derived crown radius-height models (Table 2). N means not significant, Y means significant at 95% significance level. L, M, U represent lower, middle, and upper elevational zones, respectively.

Similar results were revealed for species-specific crown radius-height models that explained around 90% of variability in crown radius data (Table 2). The models differed between the locations for each tree species except for *Salix* (Figure A4). The impact of the elevational gradient on this relationship was most profound for *Picea obovata* Ledeb. followed by *Alnus fruticosa* Rupr. (Figure A4). The crown width is a useful characteristic in studies dealing with tree competition, stand density, spacing and stocking relationships, wildlife habitat suitability models, as well as production [50]. In the past, the relationship of the crown radius to stem diameter was usually studied. The analyses showed that this relationship can be accurately described with a linear model [50,51,52]. However, nowadays remote sensing techniques are becoming the more commonly used approaches of data acquisition. Hence, the relationships to tree height, which is one of the most easily determined variables using remote sensing methods [53], may be of a greater value. The authors of [54] showed that the information about crown diameter explained 78% of the biomass variance. Other works have also supported the use of crown parameters in biomass estimation [19,55,56].

In our study, we applied regional allometric equations derived by [38,43,44] that included crown diameter as one predictor of aboveground tree biomass of thin trees. The equations applied to thick trees used DBH or a combination of DBH and height as predictors. These variables are known to provide reasonably accurate species- and region-specific biomass predictions [23,28,57]. Although diameter based allometric models are most commonly used, adding a tree height into the model usually increases the accuracy of biomass predictions [23,58,59,60]. Biomass amounts estimated in our study were comparable with quantities presented in previous works from the area around the Lama lake at similar elevations, latitudes, and longitudes [39,40,41]. However, while the cited works focused on the upper timber line, we analysed tree species biomass along the elevational gradient starting almost at the lake water level. In addition, our study contains new information on species-specific dimensions, biomass, and biomass components (living and dead), and thus enhances the knowledge about forest ecosystems of the region. Previous works from the area around the Lama lake [39,40,41] documented the most pronounced upward shift of the upper timber line on southern slopes (111 ± 74 m).

**Figure A5:**
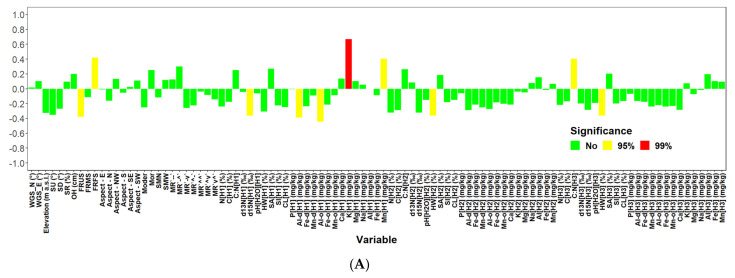

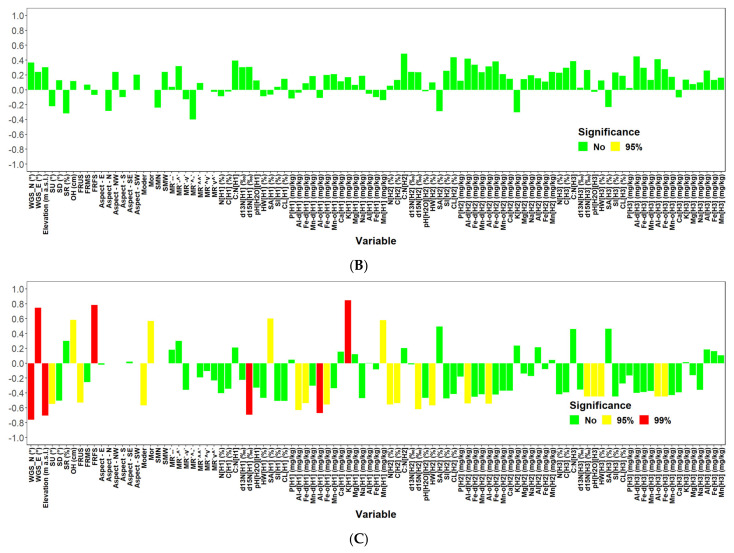
Correlation analysis of site characteristics with tree biomass (living + dead) for both locations (Keta, Lama) together (**A**), Keta (**B**) and Lama (**C**).

Our analysis revealed a significant reduction in aboveground biomass with the increasing elevation at the Lama location, which is consistent with the results of [39,41,61]. However, at the Keta location, the relationship of biomass to elevation did not have a decreasing trend but followed a U shape (Figure 3). This may be the result of the opposing impacts of elevation and micro-site conditions [62]. Topographic factors (elevation, slope, and aspect) have a significant impact on AGB (carbon) [63,64,65]. The authors of [39] confirmed a strong influence of aspect in the region of our interest, and our data showed that the aspects of the two locations differed from each other. Plots at Lama were situated on slopes with the E and SE aspect, while south was the prevailing aspect of Keta plots, followed by the western and the northern ones. Inversed impacts are known to occur around large lakes due to moisture [66] that can substantially change at a regional level because of the terrain morphology [67]. At Lama we found several significant correlations of tree biomass with site characteristics that represent either soil conditions or terrain morphology (Figure A5). Strong impacts of relief forms on forest cover were revealed by [68].

From soil characteristics, potassium content in the upper soil layer was found to be closely related to the amount of tree biomass. A high amount of potassium in soil favours the aboveground biomass production [69]. The importance of K for plant functioning was reported by, e.g., [70]. Potassium affects the stomata opening, photosynthesis, enzyme activation, and protein synthesis [71,72]. However, the relationship between tree biomass and potassium content in soil is bidirectional. On one side, a tree obtains potassium from soil. On the other hand, foliage biomass is the main source of K in soil [73]. Hence, the cycle of potassium in forest ecosystems is semi-closed. Unlike other base cations, the distribution and seasonal dynamics of K in forest ecosystems is strongly affected by biotic processes [74] because it is most prone to leaching [75]. Further fate of the leached K is driven by soil hydrologic conditions, its absorptive ability, soil microorganisms and plant roots [75]. Potassium reabsorption by plants may be higher at sites with lower percolation, i.e., where the humus layer is thicker and soil water movement caused by temperature is lower. Timber harvest, fire, land use changes, and nitrogen deposition also influence the biotic availability of potassium [76].

At Lama, two highly significant negative correlations of tree biomass to site characteristics were revealed (Figure 9). The first one was with the d15N isotope abundance in the upper soil layer (0–10 cm). The natural nitrogen stable isotope (15N) has been used in studies of N cycling in an ecosystem because its abundance is the result of a number of biogeochemical processes [77]. Nitrogen isotopes provide insights about the forms of nitrogen sources, and the processes N has been involved in [78]. Our analysis revealed higher abundances of d15N at plots with less tree biomass occurring at higher elevations. This corresponds with the knowledge that soil and plant d15N are positively correlated with mean annual temperature and negatively correlated with mean annual precipitation total [77], or the fact that in many wet or cold ecosystems d15N values in foliage and soil are depleted [79]. In multiple regression models derived for Lama, the abundance of d15N isotope in the bottom soil layer 20–30 cm was a significant predictor of tree biomass occurring in all models with three or more independent variables (Figure 9). Unlike its abundance in the first layer, the d15N abundance in the bottom layer showed a positive association with biomass in the derived models including the 7-dimensional model for Lama (Table 5). This is probably because organic matter containing N in deeper soil layers originated from trees and their roots, possibly also older and more humified than the organic matter in the topsoil (upper 10 cm), originating from shrubs that are more abundant under an open forest canopy.

Another highly significant correlation at Lama was found between the biomass and content of oxalate-extractable aluminium in the first soil layer (Figure A5). Aluminium is known for its toxicity to plants, especially if present in large amounts in acidic soils [80,81]. Measured soil pH at our locations indicated that soils were slightly to moderately acidic, while a lower pH was observed at Lama. Acidification of the location can be caused by acid rain containing emissions of Norilsk metallurgical Combine [61]. Our univariate correlation analysis revealed a negative impact of oxalate-extractable aluminium on tree biomass at Lama (Figure A5). Multiple regressions contained two other predictors representing aluminium in different soil layers, too. We found the opposing impacts of oxalate-extractable and dithionite-extractable aluminium in derived models for Lama, Keta and for both locations together (Figure 7, Figure 8 and Figure 9). In joint models, dithionite-extractable aluminium in the first soil layer, which is an indicator of more advanced weathering, had a significant positive impact on biomass. This is consistent with the study of [80] which revealed that at low concentrations aluminium can influence growth positively. The influence of aluminium oxides (dithionite and oxalate extractions) bears a witness to more intense soil weathering accompanied by a lower pH, and thus increased availability of the sorption capacity for soil organic carbon (organic matter) that in turn increases the soil capacity for nutrients.

Other highly significant variables affecting tree biomass at Lama were latitude (negative impact) and longitude (positive impact) that occurred also in multiple regression models derived for Lama (see Figure 9), while in the case of Keta only latitude was among multiple predictors (Figure 8), and in models derived for both locations together they did not occur at all (Figure 7). Although their influence is well-known and documented [82], revealing significant correlations at a micro-spatial level indicates substantial changes in site conditions even at such a small scale. Side slopes of the Putorana table mountain are steep, and the valleys are deep. At bottom parts, soils are deeper and have enough water, due to which biomass production is high there. Ecosystem productivity is also affected by soil organic matter, the stable fraction of which is humus [83]. Mor forms of humus and the thickness of O horizon were significantly positively corelated to the tree biomass at Lama, while moder forms had a negative correlation to tree biomass (Figure 7). In multiple regression models for Lama, only the thickness of the O horizon was included as a predictor, and its impact was negative indicating interrelationships between independent variables (Figure 9, Table 5). On the contrary, the models for Keta and for both locations together contained humus forms, but not the thickness of O horizon.

Soil texture has a profound influence on forest growth because it affects soil water-holding and ion exchange capacity, retention of organic matter, and aeration [71,72]. At Lama, the sand content in the first soil layer (0–10 cm) had a significant impact on tree biomass in univariate and multiple regression models (Figure A5 and Figure 9). It was also one of predictors in models derived for both locations together (Figure 7). However, while its impact at Lama was positive, in general models it was negative (Table 3 and Table 5). At Lama, more sand was found in soils at the bottom of the elevational gradient, where stands were most productive. The relationships of biomass at Lama to the content of clay, silt or hygroscopic water were negative, but the variables were not included in location-specific multiple regression models. These results reflect the more intense weathering at the upper parts of the elevational gradient because (1) the O horizon is thinner at the mountain top, due to which soil can be warmed up faster than in the bottom parts characterised by thicker layers of O horizon thermally insulating soil, (2) of strong thermal inversion along the slopes next to the lakes [66]. Models for Keta contained only clay content in the middle soil layer (10–20 cm) as a predictor (Figure 8). The percentage of hygroscopic water in the first and the third layers occurred in models for both locations, while their impact changed with model dimensions (Figure 7). Negative univariate correlations between biomass and hygroscopic water indicate that the more soil water is bound to soil particles and thus unavailable to plants [84], the less biomass is produced. In addition, the presence of hygroscopic water in soil indicates the presence of allophane or imogolite, which may limit phosphorus availability. The interrelationships are complex, some may support and some limit the growth, as shown in some of the derived multiple models, where the trend was reversed, which was documented by higher values of VIF for the parameters.

Although the C:N ratio in soil is an important characteristic of ecosystems because it affects nitrogen cycling and the process of decomposition [85] and is considered as an indicator of site quality [86], it was included only in multiple models for Keta or both locations together (Figure 7, Figure 8 and Figure 9). The univariate correlation analysis revealed that C:N ratios in all soil layers had a positive impact on tree biomass (Figure A5). However, when the variables were included in multiple regression models, only the impact of C:N ratio in the second layer remained positive, while the partial influence of the other two was negative (Figure 7 and Figure 8, Table 3 and Table 4). A similar situation was observed for the content of oxalate-extractable and dithionite-extractable iron. These variables occurred only in models derived for Keta, in which the content of dithionite-extractable iron in the second layer had a positive association, while oxalate-extractable iron in the same soil layer had a negative relationship with tree biomass (Figure 8), although the univariate regression analysis found positive correlations of both of them to tree biomass (Figure A5). Previous research showed that the shortage of Fe in soil can have a negative effect on biomass production [87], which is not the case in the analysed areas.

## 4. Materials and Methods

### 4.1. Empirical Data

**Figure 10:**
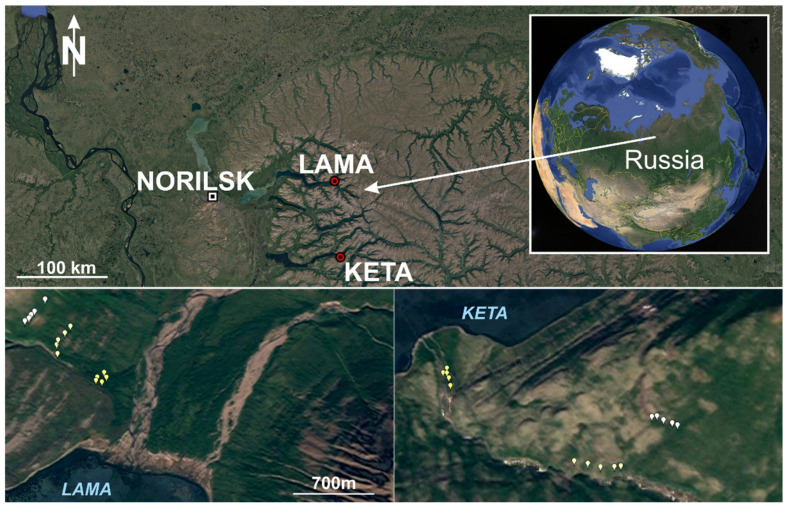
Location of sample plots in Russia, at Lama and Keta lakes (Krasnoyarsk region).

Data used for the analysis come from field measurements performed in the western part of Putorana Plateau (Figure 10) in the area around the Lama and Keta lakes (Krasnoyarsk region, Russia) in the years 2018 and 2019. This part of northern Russia is one of the least accessible and least studied regions. The mountain range represents the biggest monolithic mountain range of the Russian Arctic, which is almost completely located north of the arctic circle [39,61] extending from 89° to 101° E and from 67° to 71° N. The geological substrate is the tholeiitic basalt characterised by homogeneous chemical and mineralogical composition with high contents of Al_2_O_3_ and Fe_2_O_3_ [88]. With respect to the geological and geomorphological features, it is a flat-topped basalt crystalline massif (plateau) with elevations averaging 900–1200 m and reaching a maximum of 1701 m a.s.l. in the central part (Kamen Mountain). Multiple uplifts on the Putorana Plateau have generated deep radial tectonic fractures in this area in the form of narrow gorges and canyons with the trappean structure of slopes [39,41,68]. The Putorana Plateau is in the subarctic climate belt, at the boundary between the Atlantic and Siberian regions, in the continuous permafrost zone. The climate is excessively continental [88], and the amount of precipitation is significantly higher than anywhere else in the north of Eastern Siberia. The cold subarctic climate sustains the presence of continuous permafrost table at 1 to 3 m soil depth and a widespread development of cryogenic processes, e.g., solifluction [89].

The unique geological formation of Putorana Plateau was the main reason for assigning this region to the World Natural Heritage in 2010 [90]. The area is characterised by the occurrence of unique natural complexes with high biodiversity and the presence of intrazonal communities of flora and fauna. The upper timberline is formed by Dahurian larch (*Larix gmelinii* Rupr.) and lies in the interval of 200 to 900 m a.s.l., depending on regional and local habitat conditions.

At each location we gathered data along an elevational gradient divided into three elevational zones. In every elevational zone we established five circular sample plots, each of 500 m^2^ situated along the same contour line. Hence, in total we established 30 sample plots. More than 150 characteristics describing site, terrain, soil, ecological, and tree species-specific features, were assessed in the field at each plot. The tree species was defined as a species of a tree shape with a potential tree height of 5 m or more. Plot position and elevation were measured with a GPS tool Garmin 60CSx. Aspect and slope were measured in degrees and were determined with a compass and Hypsometer Haglöf Vertex, respectively. Relief forms were specified visually based on the plot position along the slope and with regard to the prevailing water movement (predominant water inflow, outflow, or balanced). We identified five forms of relief: flat terrain, peak/upper slope, middle slope, foot of slope, and indistinct. Micro-relief was evaluated based on the terrain curvature along the contour line and along the slope using three basic terrain shapes: - flat land, ^ concave terrain, ^v^ convex terrain. Hence, 9 possible combinations were identified in total, e.g., -^ is flat land along the contour line and concave terrain along the slope. Then, we assessed the form of humus, estimated moisture conditions (dry, optimal, wet sites), relative coverage of aboveground rocks, and of fine woody debris (%). Mean diameter of fine woody debris, diameter, and length of coarse woody debris (CWD) pieces were measured. Each CWD piece was assigned into one of four decay classes (1—recently dead wood with intact bark, present twigs and branches, round shape, smooth surface, intact texture, and the position elevated on support points, 2—solid wood with present bark and twigs with diameter over 1 cm, 3—moderately soft wood, twigs absent, bark mostly absent, 4—very soft deadwood with no bark, twigs or branches, strongly fragmented, in contact with the ground along the whole length). In total, we gathered information about 240 pieces of deadwood. In the case of living trees, we collected information about individual tree species for four categories: trees with diameter at breast height (DBH) above 7 cm, trees with tree height exceeding 1.3 m and DBH below 7 cm, trees with a height from 0.2 to 1.3 m, and trees smaller than 0.2 m). For each category we determined tree species cover, number of trees, mean tree height, mean crown radius, and minimum, maximum, and mean diameter at breast height where applicable. In total, we measured 576 trees with tree height exceeding 1.3 m. The crown radius was measured at 382 trees representing all above-listed size categories. The basic description of sample plots sites is presented in Table 6, while the basic characteristics of individual tree species are shown in Table 7.

### 4.2. Soil Sampling and Analyses

Soil sampling was performed at each research plot, i.e., 30 soil pits were excavated to the depth of 0.3–0.4 m from the mineral soil surface in total. Three soil samples, each weighing approximately 150 g, were taken from 0.1 m, 0.2 m, and 0.3 m depths at each plot. The soil texture was determined by the pipette method after the removal of organic compounds with hydrogen peroxide (H_2_O_2_) and clay dispersion [92]. The soil pH was measured in a deionized water suspension of air-dried soil at a soil-to-solution ratio of 1:2.5 by Testo 206 pH1 instrument (Testo SE & Co. KGaA, Lenzkirch, Germany). Contents of C and N in the fine earth (<2 mm) were obtained through dry combustion method using CN analyser (Vario Isotope Cube, Elementar Analysis Systems GmbH, Hanau, Germany). The C:N ratio was calculated as an indicator of POM presence (>10), as opposed to minerally associated organic matter (<13) [93]. Free Fe (Fe-o, Fe-d) and Al (Al-o, Al-d) contained in the fine earth fraction were extracted using 0.2 M ammonium oxalate and sodium dithionite-citrate solutes [94]. Dithionite-citrate extraction represents both crystalline and poorly crystalline Fe oxides [95,96]. The value of Al d is thus believed to represent the amount of Al substituted in Fe oxides and hydroxides [97], whereby Al substitution in iron oxides indicates stronger weathering conditions [98]. Oxalate-extractable Fe, Al, and Si represent poorly crystalline aluminosilicates, ferrihydrite, and Al and Fe in organic complexes [95,96]. Barium chloride (BaCl_2_), 0.1 M, was used to extract exchangeable Ca, Mg, and K [94]. Free Fe, Al, and exchangeable cations concentrations were measured by inductively coupled plasma—optical emission spectrometer (ICP—OES Agilent 5100, USA).

### 4.3. Statistical Analyses

Standard statistical approaches were applied (univariate and multivariate correlation and regression analyses, ANOVA) to perform the analyses using the available tools in R environment [99]. We used the nls function in R to derive non-linear height-diameter and crown radius-height models. For multiple analyses we used the lmSubsets function [100], and the ggplot2 package to visualise the results [101].

### 4.4. Non-Linear Height-Diameter Models and Crown Radius-Height Models of Trees

Tree height (h) is the second most important variable after the diameter at breast height (DBH) because it describes a vertical stand structure and serves as a good indicator of site productivity [102]. The relationship between tree diameter and tree height is related to the growth stage, i.e., its position and shape changes with stand age. This is caused by varying growth rates of height and diameter in individual developmental stages of stands. The height curve of natural forests is usually in a balanced state, i.e., its position and shape do not change. Literature sources present a number of functions suitable for modelling the relationship between tree height and diameter [15,48,49,103,104,105,106,107,108]. In our study we applied a slightly modified Prodan function [48,49] in the following form:

(1) $$h=\frac{DBH^{2}}{a+b\times DBH+DBH^{2}}+1.3$$

where *h* is tree height in m, *DBH* is tree diameter in cm, *a* and *b* are regression coefficients.

Tree crown size is another important parameter that affects tree growth, carbon sequestration, shading, filtering of fine air particulates, and risk of windbreaking [52]. The majority of allometric relationships between crown diameter, radius, or crown projection use tree diameter as a predictor. However, currently, remote sensing techniques have become to be more frequently used. Therefore, we decided to take a tree height as an explanatory variable since height can be directly obtained from remote sensing data. Moreover, such a relationship can be also applied to trees that did not reach a height of 1.3 m yet. To describe the relationship between the crown radius (*CR*) and tree height (*h*) we used the following nonlinear equation:

(2) $$CR=\frac{h}{a\cdot h+b}$$

where *CR* is crown radius in m, *h* is tree height in m, *a* and *b* are regression coefficients.

### 4.5. Quantifying the Above-Ground Biomass (AGB)

Biomass plays an important role in biogeochemical cycles, ecosystem functioning, and the formation of community structure [57]. For the quantification of the biomass of individual tree species recorded in our study we used previously published models for the total aboveground biomass of living trees including stem, branches, twigs, and foliage, from similar geographical regions [38,43,44]. The aboveground biomass of trees with DBH above 7 cm was calculated using DBH as a sole predictor (*Picea*), or using both DBH and height (*Larix, Betula, Alnus, Salix, Sorbus*). In the case of smaller trees that did not reach DBH of 7 cm or a height of 1.3 m, we calculated the aboveground biomass as a function of tree height and crown diameter.

The biomass amount of deadwood was calculated for individual components: aboveground coarse deadwood, buried deadwood, standing dead trees, snags, stumps, fine woody debris. First, the volume of deadwood was determined as follows. The volume of aboveground deadwood and buried deadwood pieces was calculated using Huber formula based on their middle stem diameter and length [102,109]. The volume of standing dead trees or snags was quantified using the standard volume equation with DBH, height, and a taper factor [110] as independent variables, while the taper factor was set to 0.4 [102]. The volume of stumps was calculated as a volume of a cylinder using its diameter and height. The volume of fine woody debris was derived from its cover area and mean diameter. In the next step, volume was converted to biomass using wood density. We set the mean wood density of living trees to 400 kg/m^3^ and reduced it with regard to decay stage following the work of [111]. Hence, the density of wood in decay stage 1, 2, 3, and 4 represented 90%, 75%, 50%, and 35% of the living wood density, respectively. In the case of buried deadwood it was only 20%.

## 5. Conclusions

The presented study brings new information and knowledge about tree vegetation from a part of northern Russia that is one of the least accessible and least studied regions. This study is the first one focusing on the area around Keta lake. The developed local height-diameter and crown radius-height models for six tree species (*Larix gmelinii* (Rupr.), *Picea obovata* Ledeb., *Alnus fruticosa* Rupr., *Salix jenisseensis* (F. Schmidt) Flod., *Betula tortuosa* Ledeb., *Sorbus sibirica* Hedl.) were site and elevation-specific, and explained most of the variability in studied relationships. They may help to improve estimates of biomass and carbon stock in the studied regions in the future. The total tree biomass stock including aboveground tree biomass and aboveground and buried deadwood varied between 6.47 t/ha and 149 t/ha, while the total deadwood biomass fluctuated from 0.06 to 21.45 t/ha. The general pattern of decreasing biomass production with elevation was observed at Lama. At the Keta location, the relationship of biomass to elevation followed a U shape. This may be the result of the opposing impacts of elevation and micro-site conditions.

Terrain morphology and soil conditions had a significant impact on tree biomass. The most influential predictors in multiple regression models for both locations together were the form of relief representing the peak, and upper slope, followed by elevation, the form of relief representing the foot of slope, ratio of stable nitrogen isotopes 15N:14N in soil depth of 20–30 cm, and C:N ratio in soil depth of 10–20 cm. However, the multiple models derived for individual locations separately differed in most of predictors they contained. Hence, the results indicate that microsite environmental conditions have a substantial effect on tree biomass in the studied locations. Further detailed research is needed to thoroughly understand the functioning and dynamics in this part of the world.

## Figures and Tables

**Table 1:** Statistical parameters of derived height-diameter models (Model—identification number of the derived model, PAR—coefficient in Equation (1), COEF—value of the coefficient in Equation (1), SE—standard error, *t* value—*t*-value of Student test of a coefficient, *p* value—probability of *t*-value and its significance as follows: .—90%, *—95%, **—99%, ***—99.9%, R^2^—R-squared; Level: elevational zone L—Lower, M—Middle, U—Upper, Number—number of trees).

Model	PAR	COEF	SE	*t* Value	*p* Value		R^2^	Tree Species	Location	Level	Number
1	a	0.690	0.164	4.2	0.00	**	0.97	*Alnus fruticosa* Rupr.	Keta	L	15
	b	0.205	0.033	6.2	0.00	***					
2	a	1.198	0.164	7.3	0.00	***	0.97	*Alnus fruticosa* Rupr.	Keta	U	15
	b	0.077	0.032	2.4	0.03	*					
3	a	1.116	0.176	6.3	0.00	***	0.97	*Alnus fruticosa* Rupr.	Keta	M	12
	b	0.091	0.032	2.8	0.02	*					
4	a	1.558	0.309	5.1	0.00	***	0.88	*Alnus fruticosa* Rupr.	Lama	L	30
	b	0.130	0.036	3.6	0.00	**					
5	a	0.918	0.315	2.9	0.01	*	0.93	*Alnus fruticosa* Rupr.	Lama	U	15
	b	0.320	0.074	4.3	0.00	**					
6	a	1.215	0.310	3.9	0.00	**	0.93	*Alnus fruticosa* Rupr.	Lama	M	21
	b	0.174	0.046	3.8	0.00	**					
7	a	1.163	0.193	6.0	0.00	***	0.88	*Betula tortuosa* Ledeb.	Keta	U	24
	b	0.041	0.015	2.8	0.01	*					
8	a	1.003	0.134	7.5	0.00	***	0.88	*Betula tortuosa* Ledeb.	Keta	M	27
	b	0.040	0.012	3.5	0.00	**					
9	a	1.274	0.170	7.5	0.00	***	0.91	*Betula tortuosa* Ledeb.	Lama	U	30
	b	−0.008	0.014	−0.6	0.57						
10	a	1.607	0.164	9.8	0.00	***	0.95	*Betula tortuosa* Ledeb.	Lama	M	24
	b	0.016	0.008	1.9	0.07	.					
11	a	1.453	0.270	5.4	0.00	***	0.97	*Larix gmelinii* (Rupr.)	Keta	L	18
	b	0.018	0.007	2.4	0.03	*					
12	a	0.841	0.111	7.6	0.00	***	0.96	*Larix gmelinii* (Rupr.)	Keta	U	24
	b	0.038	0.004	8.7	0.00	***					
13	a	0.951	0.138	6.9	0.00	***	0.96	*Larix gmelinii* (Rupr.)	Keta	M	21
	b	0.041	0.006	6.3	0.00	***					
14	a	0.832	0.127	6.6	0.00	***	0.92	*Larix gmelinii* (Rupr.)	Lama	L	18
	b	0.021	0.004	4.9	0.00	***					
15	a	0.851	0.093	9.2	0.00	***	0.97	*Larix gmelinii* (Rupr.)	Lama	U	24
	b	0.057	0.004	14.0	0.00	***					
16	a	0.937	0.130	7.2	0.00	***	0.94	*Larix gmelinii* (Rupr.)	Lama	M	27
	b	0.023	0.005	4.9	0.00	***					
17	a	1.421	0.107	13.3	0.00	***	0.99	*Picea obovata* Ledeb.	Keta	L	27
	b	0.014	0.003	4.8	0.00	***					
18	a	1.280	0.103	12.5	0.00	***	0.97	*Picea obovata* Ledeb.	Keta	U	30
	b	0.024	0.004	5.6	0.00	***					
19	a	1.303	0.168	7.8	0.00	***	0.95	*Picea obovata* Ledeb.	Keta	M	24
	b	0.020	0.006	3.1	0.01	**					
20	a	1.238	0.230	5.4	0.00	***	0.92	*Picea obovata* Ledeb.	Lama	L	24
	b	0.011	0.008	1.4	0.19						
21	a	1.508	0.227	6.6	0.00	***	0.90	*Picea obovata* Ledeb.	Lama	U	21
	b	0.028	0.010	2.9	0.01	*					
22	a	1.329	0.196	6.8	0.00	***	0.90	*Picea obovata* Ledeb.	Lama	M	30
	b	0.017	0.009	1.9	0.06	.					
23	a	0.973	0.082	11.9	0.00	***	1.00	*Salix jenisseensis* (F. Schmidt) Flod.	Keta	L	6
	b	0.057	0.008	7.2	0.00	**					
24	a	2.124	0.453	4.7	0.00	***	0.92	*Salix jenisseensis* (F. Schmidt) Flod.	Keta	U	15
	b	−0.034	0.077	−0.4	0.67						
25	a	2.645	0.894	3.0	0.02	*	0.92	*Salix jenisseensis* (F. Schmidt) Flod.	Keta	M	9
	b	−0.372	0.382	−1.0	0.36						
26	a	0.237	0.643	0.4	0.73		0.63	*Salix jenisseensis* (F. Schmidt) Flod.	Lama	U	6
	b	0.563	0.185	3.0	0.04	*					
27	a	−0.255	0.300	−0.9	0.44		0.14	*Salix jenisseensis* (F. Schmidt) Flod.	Lama	M	6
	b	0.338	0.054	6.2	0.00	**					
28	a	1.340	0.429	3.1	0.01	**	0.90	*Sorbus sibirica* Hedl.	Keta	L	18
	b	0.088	0.058	1.5	0.15						
29	a	0.964	0.334	2.9	0.05	*	0.97	*Sorbus sibirica* Hedl.	Lama	L	6
	b	0.165	0.093	1.8	0.15						
General models											
1	a	1.063	0.117	9.1	0.00	***	0.92	*Alnus fruticosa* Rupr.	All	All	108
	b	0.184	0.017	10.8	0.00	***					
2	a	1.075	0.083	12.9	0.00	***	0.87	*Betula tortuosa* Ledeb.			111
	b	0.034	0.006	5.6	0.00	***					
3	a	0.994	0.090	11.1	0.00	***	0.88	*Larix gmelinii* (Rupr.)			132
	b	0.028	0.003	9.0	0.00	***					
4	a	1.391	0.078	17.9	0.00	***	0.93	*Picea obovata* Ledeb.			156
	b	0.015	0.003	5.5	0.00	***					
5	a	1.879	0.205	9.2	0.00	***	0.89	*Salix jenisseensis* (F. Schmidt) Flod.			42
	b	−0.007	0.021	−0.3	0.75						
6	a	1.372	0.247	5.6	0.00	***	0.92	*Sorbus sibirica* Hedl.			27
	b	0.083	0.035	2.4	0.03	*					

**Table 2:** Statistical parameters of derived crown radius-height models (Model—identification number of the derived model, PAR—coefficient in Equation (2), COEF—value of the coefficient in Equation (2), SE—standard error, *t* value—*t*-value of Student’s test of coefficient, *p* value—probability of *t* value and its significance as follows: .—90%, *—95%, **—99%, ***—99.9%, R^2^—R-squared; Level: elevational zone L—Lower, M—Middle, U—Upper, Number—number of trees).

Model	PAR	COEF	SE	*t* Value	*p* Value		R^2^	Tree Species	Location	Level	Number
1	a	1.651	0.100	15.8	0.00	***	0.95	*Alnus fruticosa* Rupr.	Keta	L	15
	b	0.815	0.130	6.3	0.00	***					
2	a	1.627	0.130	12.6	0.00	***	0.96	*Alnus fruticosa* Rupr.	Keta	U	15
	b	1.229	0.170	7.1	0.00	***					
3	a	1.167	0.100	11.1	0.00	***	0.97	*Alnus fruticosa* Rupr.	Keta	M	12
	b	1.086	0.150	7.2	0.00	***					
4	a	0.010	0.160	0.1	0.95		0.94	*Alnus fruticosa* Rupr.	Lama	L	20
	b	4.036	0.640	6.3	0.00	***					
5	a	1.338	0.230	5.8	0.00	***	0.86	*Alnus fruticosa* Rupr.	Lama	U	14
	b	1.389	0.340	4.1	0.00	**					
6	a	0.171	0.210	0.8	0.44		0.88	*Alnus fruticosa* Rupr.	Lama	M	15
	b	3.368	0.690	4.9	0.00	***					
7	a	0.225	0.150	1.5	0.16		0.88	*Betula tortuosa* Ledeb.	Keta	U	15
	b	4.419	1.060	4.2	0.00	**					
8	a	0.244	0.130	1.9	0.08	.	0.84	*Betula tortuosa* Ledeb.	Keta	M	17
	b	3.749	0.920	4.1	0.00	**					
9	a	0.378	0.050	7.8	0.00	***	0.95	*Betula tortuosa* Ledeb.	Lama	U	21
	b	2.468	0.330	7.6	0.00	***					
10	a	0.335	0.050	6.6	0.00	***	0.96	*Betula tortuosa* Ledeb.	Lama	M	15
	b	2.793	0.340	8.1	0.00	***					
11	a	0.281	0.020	14.1	0.00	***	0.99	*Larix gmelinii* (Rupr.)	Keta	L	16
	b	2.255	0.290	7.7	0.00	***					
12	a	0.177	0.060	2.9	0.01	*	0.97	*Larix gmelinii* (Rupr.)	Keta	U	15
	b	3.521	0.810	4.4	0.00	**					
13	a	0.282	0.060	4.8	0.00	***	0.96	*Larix gmelinii* (Rupr.)	Keta	M	15
	b	2.525	0.610	4.2	0.00	**					
14	a	0.329	0.050	6.5	0.00	**	0.66	*Larix gmelinii* (Rupr.)	Lama	L	6
	b	1.701	0.800	2.1	0.10						
15	a	0.180	0.030	6.4	0.00	***	0.99	*Larix gmelinii* (Rupr.)	Lama	U	13
	b	2.657	0.280	9.4	0.00	***					
16	a	0.166	0.030	5.2	0.00	***	0.97	*Larix gmelinii* (Rupr.)	Lama	M	13
	b	3.368	0.490	6.9	0.00	***					
17	a	0.494	0.030	19.0	0.00	***	0.98	*Picea obovata* Ledeb.	Keta	L	19
	b	2.562	0.260	10.0	0.00	***					
18	a	0.512	0.060	8.5	0.00	***	0.93	*Picea obovata* Ledeb.	Keta	U	20
	b	2.782	0.500	5.6	0.00	***					
19	a	0.627	0.040	17.3	0.00	***	0.97	*Picea obovata* Ledeb.	Keta	M	18
	b	2.353	0.260	9.1	0.00	***					
20	a	0.428	0.020	25.5	0.00	***	0.99	*Picea obovata* Ledeb.	Lama	L	15
	b	2.597	0.200	12.9	0.00	***					
21	a	0.351	0.070	4.9	0.00	**	0.93	*Picea obovata* Ledeb.	Lama	U	11
	b	3.317	0.600	5.6	0.00	***					
22	a	0.324	0.040	8.0	0.00	***	0.97	*Picea obovata* Ledeb.	Lama	M	16
	b	3.312	0.350	9.5	0.00	***					
23	a	0.441	0.190	2.4	0.14		0.97	*Salix jenisseensis* (F. Schmidt) Flod.	Keta	L	4
	b	3.389	1.040	3.3	0.08	.					
24	a	1.312	0.170	7.6	0.00	***	0.91	*Salix jenisseensis* (F. Schmidt) Flod.	Keta	U	10
	b	1.359	0.280	4.8	0.00	**					
25	a	1.444	0.440	3.3	0.08	.	0.58	*Salix jenisseensis* (F. Schmidt) Flod.	Keta	M	4
	b	0.997	0.680	1.5	0.28						
26	a	0.707	0.140	5.2	0.12		0.99	*Salix jenisseensis* (F. Schmidt) Flod.	Lama	M	3
	b	3.052	0.530	5.8	0.11						
27	a	0.182	0.340	0.5	0.61		0.68	*Sorbus sibirica* Hedl.	Keta	L	9
	b	3.888	1.400	2.8	0.03	*					
28	a	0.826	0.140	6.0	0.01	**	0.97	*Sorbus sibirica* Hedl.	Lama	L	5
	b	1.925	0.290	6.7	0.01	**					
29	a	0.993	0.150	6.5	0.02	*	0.99	*Sorbus sibirica* Hedl.	Lama	M	4
	b	1.296	0.210	6.1	0.03	*					
General models											
1	a	0.370	0.077	4.8	0.00	***	0.86	*Alnus fruticosa* Rupr.	All	All	91
	b	2.855	0.221	12.9	0.00	***					
2	a	0.317	0.041	7.7	0.00	***	0.9	*Betula tortuosa* Ledeb.			71
	b	3.174	0.292	10.9	0.00	***					
3	a	0.249	0.016	15.8	0.00	***	0.96	*Larix gmelinii* (Rupr.)			78
	b	2.547	0.208	12.2	0.00	***					
4	a	0.460	0.018	24.9	0.00	***	0.95	*Picea obovata* Ledeb.			99
	b	2.778	0.170	16.3	0.00	***					
5	a	0.743	0.109	6.8	0.00	***	0.85	*Salix jenisseensis* (F. Schmidt) Flod.			23
	b	2.431	0.308	7.9	0.00	***					
6	a	0.601	0.159	3.8	0.00	***	0.79	*Sorbus sibirica* Hedl.			20
	b	2.298	0.471	4.9	0.00	***					

**Table 3:** Overview of variables included in the optimal multiple regression model (Biomass = f(Variable1,…, Variable13)) derived for Keta and Lama together and their statistical characteristics (H1—layer 0–10 cm, H2—layer 10–20 cm, H3—layer 20–30 cm, MR`-^`—Micro relief—`-^`, HW (%)—hygroscopic water, SA (%)—sand fraction, C:N—C:N ratio, C (%)—carbon content, d15N (‰)—ratio of stable nitrogen isotopes 15N:14N, Elevation (m a.s.l.)—elevation, FRUS—form of relief (peak, upper slope), FRFS—form of relief(foot of slope), SD (°)—slope downward, Estimate = regression coefficient for the respective variable, SE= standard error, *t* value—*t*-value of Student test of coefficient, *p* value—probability of *t* value and its significance of *p* value as follows: *—95%, **—99%, ***—99.9%, VIF—variance inflation factor, Fdf—degrees of freedom, Fp—*p* value for F, R^2^adj—adjusted R-squared).

PC	Variable	Estimate	SE	*t* Value	*p* Value		VIF	FModel	Fdf1	Fdf2	Fp	R^2^adj
1	(Intercept)	−333.02	37.28	−8.93	7.9 × 10^−8^	***		21.76	12	17	7.7 × 10^−8^	0.90
2	FRFS	215.47	19.99	10.78	5.1 × 10^−9^	***	4.53					
3	FRUS	−135.54	14.73	−9.20	5.2 × 10^−8^	***	2.64					
4	Elevation (m a.s.l.)	0.75	0.08	8.94	7.7 × 10^−8^	***	4.45					
5	C:N[H2]	19.04	2.17	8.77	1.0 × 10^−7^	***	2.77					
6	MR`-^`	47.86	6.05	7.91	4.3 × 10^−7^	***	1.44					
7	C:N[H3]	−14.91	2.28	−6.55	4.9 × 10^−6^	***	2.67					
8	HW[H1] (%)	12.99	2.20	5.91	1.7 × 10^−5^	***	1.76					
9	d15N[H3] (‰)	40.26	7.69	5.23	6.8 × 10^−5^	***	5.91					
10	d15N[H2] (‰)	−37.72	7.72	−4.89	1.4 × 10^−4^	***	6.36					
11	SD (°)	0.84	0.25	3.36	3.7 × 10^−3^	**	1.99					
12	SA[H1] (%)	−0.81	0.24	−3.36	3.7 × 10^−3^	**	1.53					
13	C[H2] (%)	−4.09	1.66	−2.47	2.5 × 10^−2^	*	2.00					

**Table 4:** Overview of variables included in the optimal multiple regression model (Biomass = f(Variable1, …, Variable5)) derived for Keta and their statistical characteristics (H1—soil layer 0–10 cm, H2—layer 10–20 cm, H3—layer 20–30 cm, C:N—C:N ratio, WGS_N (°)—latitude, CL (%)—clay fraction, Estimate—regression coefficient for the respective variable, SE—standard error, *t* value—*t*-value of Student test of coefficient, *p* value—probability of *t* value and its significance of *p* value as follows: .—90%,*—95%, **—99%, ***—99.9%, VIF—variance inflation factor, Fdf—degrees of freedom, Fp— *p* value for F, R^2^adj—adjusted R-squared).

PC	Variable	Estimate	SE	*t* Value	*p* Value	pSig	VIF	FModel	Fdf1	Fdf2	Fp	R^2^adj
1	(Intercept)	−58,314.23	21,520.15	−2.71	0.02	*		5.22	4	10	0.02	0.55
2	C:N[H2]	26.83	8.70	3.08	0.01	*	2.46					
3	WGS_N (°)	844.68	312.71	2.70	0.02	*	1.08					
4	CL[H2] (%)	6.37	2.59	2.46	0.03	*	1.06					
5	C:N[H3]	−11.57	5.25	−2.21	0.05	.	2.52					

**Table 5:** Overview of variables included in the optimal multiple regression model (Biomass = f(Variable1, …, Variable7)) derived for Lama and their statistical characteristics (H1—soil layer 0–10 cm, H2—layer 10–20 cm, H3—layer 20–30 cm, d15N (‰)—ratio of stable nitrogen isotopes 15N:14N, K (mg/kg)—potassium content, WGS_N (°)—latitude, OH (cm)—thickness of O horizon—surface organic layer, SA (%)—sand fraction, Estimate = regression coefficient for the respective variable, SE= standard error, *t* value—*t*-value of Student test of coefficient, *p* value—probability of *t* value and its significance of *p* value as follows: .—90%, *—95%, **—99%, ***—99.9%, VIF—variance inflation factor, Fdf—degrees of freedom, Fp—*p* value for F, R^2^adj—adjusted R-squared).

PC	Variable	Estimate	SE	*t* Value	*p* Value	pSig	VIF	FModel	Fdf1	Fdf2	Fp	R^2^adj
1	(Intercept)	849,370.09	330,960.03	2.57	0.03	*		14.04	6	8	0.03	0.85
2	d15N[H1] (‰)	−21.43	6.04	−3.54	0.01	**	3.17					
3	d15N[H3] (‰)	30.29	9.38	3.23	0.01	*	3.91					
4	K[H1] (mg/kg)	0.79	0.30	2.59	0.03	*	1.72					
5	WGS_N (°)	−12,224.91	4763.32	−2.57	0.03	*	3.26					
6	OH (cm)	−6.72	3.01	−2.23	0.06	.	2.29					
7	SA[H1] (%)	1.01	0.50	2.00	0.08	.	1.41					

**Table 6:** Basic characteristics of sample plots. Mean annual temperature (MAT) and mean annual precipitation total (MAP) were calculated from the Version 4 of the CRU TS Monthly High-Resolution Gridded Multivariate Climate Dataset [91].

Location	Number of Plots	Min-Max WGS_N (°)	Min–Max WGS_E (°)	Min-Max Elevation above Sea Level (m a.s.l.)	Min-Max Slope (°)	^1^ Min-Max Aspect (°)	^2^ Min–Max Cover (%)	^3^ MAT (°C)	^4^ MAP (mm)	Geol. Substrate	Soil
Keta	15	68.75–68.76	91.49–91.55	102–351	2–24	170–350	11–70	–10.1	456.7	Basalt	Eutric Cambisol
Lama	15	69.48–69.49	91.42–91.45	111–441	6–61	70–150	15–80	–9.4	435.3	Basalt	Eutric Cambisol

Note: ^1^—azimuth, ^2^—vertical projection coverage of trees with diameter at breast height outside bark exceeding 7 cm, ^3^—mean annual temperature, ^4^—mean annual precipitation total.

**Table 7:** Basic characteristics of tree species.

Location	Tree Species	Min–Max Cover (%)	Max DBH (cm)	Max Height (m)	Min–Max Number of Trees per ha (pcs/ha)	Min–Max Basal Area (m^2^/ha)	Min–Max AGB (t/ha)
Keta	*Alnus fruticosa* Rupr.	3–101	7	5	580–21800	0–3.27	0.06–3.5
Keta	*Larix gmelinii* (Rupr.)	0.5–51.2	46	22	220–660	0–18.62	0–85.73
Keta	*Picea obovata* Ledeb.	1.2–27.5	44	23	240–1880	0.08–16.38	0.14–68.31
Keta	*Salix jenisseensis* (F. Schmidt) Flod.	0–35.5	14	8.8	0–3900	0–3.74	0–6.08
Keta	*Betula tortuosa* Ledeb.	0–37	24	12.4	0–4460	0–4.07	0–10.27
Keta	*Sorbus sibirica* Hedl.	0–14	10	5.5	0–2540	0–0.76	0–0.77
Lama	*Alnus fruticosa* Rupr.	3.2–72.8	13	6	100–15,100	0.09–5.34	0.22–7.28
Lama	*Larix gmelinii* (Rupr.)	3.9–63.6	43	26	20–540	0.63–21.28	1.91–98.06
Lama	*Picea obovata* Ledeb.	0.3–23.1	40	24	20–1220	0.04–11.79	0.04–49.45
Lama	*Salix jenisseensis* (F. Schmidt) Flod.	0–3.4	8	5	0–220	0–0.54	0–0.52
Lama	*Betula tortuosa* Ledeb.	0–88.3	30	14	0–5460	0–7.31	0–15.63
Lama	*Sorbus sibirica* Hedl.	0–1.5	4	3.5	0–240	0–0.04	0–0.06

Note: AGB—aboveground biomass calculated using equations of [38,43,44].

## Data Availability

The data presented in this study are available on request from the corresponding author. The data are not publicly available due to the ongoing analyses.

## Abbreviations

WGS_N—latitude, WGS_E—longitude, Elevation—elevation, SU—slope upward, SD—slope downward, SR—surface rockiness, OH—thickness of O horizon—surface organic layer, FRUS—form of relief (peak, upper slope), FRMS—form of relief (middle slope), FRFS—form of relief (foot of slope), Aspect—E, Aspect—N—, Aspect—NW—, Aspect—S—, Aspect—SE—, Aspect—SW—, Moder—humus, Moder—form, Mor—humus form, SMN—soil moisture normal, SMW—soil moisture wet, MR`--`, `-^`, `-v`, `^-`, `^^`, `^v`, `v^`—micro relief (combinations of terrain curvature along the contour line and along the slope using three basic terrain shapes: - flat land, ^ concave terrain, ^v^ convex terrain), H1—soil layer 0–10 cm, H2—layer 10–20 cm, H3—layer 20–30 cm, N—nitrogen content, C—carbon content, C:N—C:N ratio, d13N—ratio of stable carbon isotopes 13C:12C, d15N—ratio of stable nitrogen isotopes 15N:14N, pH[H2O]—power of hydrogen in soil, HW—hygroscopic water, SA—sand fraction, SI—silt fraction, CL—clay fraction, P—phosphorus content, Al-d—content of dithionite-extractable aluminium, Fe-d—content of dithionite-extractable iron, Mn-d—content of dithionite-extractable manganese, Al-o—content of oxalate-extractable aluminium, Fe-o—content of oxalate-extractable iron, Mn-o—content of oxalate-extractable manganese, Ca—calcium content, K—potassium content, Mg—magnesium content, Na—sodium content, Al—aluminium content, Fe—iron content, Mn—manganese content.
